# Drug Targeting and Nanomedicine: Lessons Learned from Liver Targeting and Opportunities for Drug Innovation

**DOI:** 10.3390/pharmaceutics14010217

**Published:** 2022-01-17

**Authors:** Anna Salvati, Klaas Poelstra

**Affiliations:** Department of Nanomedicine and Drug Targeting, Groningen Research Institute of Pharmacy (GRIP), University of Groningen, A. Deusinglaan 1, 9713 AV Groningen, The Netherlands

**Keywords:** drug targeting, nanomedicine, liver fibrosis, anti-fibrotic, antitumor, nanoparticle corona, mechanism of uptake, intracellular trafficking

## Abstract

Drug targeting and nanomedicine are different strategies for improving the delivery of drugs to their target. Several antibodies, immuno-drug conjugates and nanomedicines are already approved and used in clinics, demonstrating the potential of such approaches, including the recent examples of the DNA- and RNA-based vaccines against COVID-19 infections. Nevertheless, targeting remains a major challenge in drug delivery and different aspects of how these objects are processed at organism and cell level still remain unclear, hampering the further development of efficient targeted drugs. In this review, we compare properties and advantages of smaller targeted drug constructs on the one hand, and larger nanomedicines carrying higher drug payload on the other hand. With examples from ongoing research in our Department and experiences from drug delivery to liver fibrosis, we illustrate opportunities in drug targeting and nanomedicine and current challenges that the field needs to address in order to further improve their success.

## 1. Introduction

Nanosized drug carriers (nanomedicines) and smaller drug targeting constructs are used in the field of advanced drug delivery systems to improve the delivery of drugs to the target site. They can achieve this via different mechanisms and, if combined, these two strategies are an extremely powerful tool for future innovative therapies. Nanomedicines are typically larger constructs of variable size (between 10–500 nm) and composition (polymers, lipids or inorganic components, for instance, gold or silica) that can accommodate large quantities of therapeutic entities [1,2,3]. Drug delivery to the desired site of action is favored by a prolonged circulation time in blood, which is achieved via different mechanisms, such as for instance by the prevention of renal clearance through size increase and by protecting the therapeutic compounds from degradation in plasma through shielding. A prolonged circulation time favors the accumulation in the target area. In the case of tumor targeting, this is often achieved by the so-called enhanced permeability and retention (EPR) effect, where the nanomedicine passively accumulates into the tumor tissue by diffusion through leaky tumor blood vessels [4,5]. Similar EPR effects can be exploited also to target inflamed areas and other diseases characterized by leaky blood vessels [6]. Recent findings however have challenged our current understanding on the mechanisms of nanoparticle accumulation into tumors and suggested that this mainly occurs via specific active uptake mechanisms into tumor endothelial cells and later transcytosis into the tumor tissue [6,7,8]. At the same time, nanoparticles may also adsorb plasma components that may induce accumulation in the target area. Pegylated liposomal doxorubicin (Doxil) [7,9] is an example of so-called passively targeted drug exploiting the EPR effect and one of the first nanomedicines approved for clinical use (1995), while Onpattro [10,11,12] that adsorbs apolipoprotein E (apoE) in plasma and is then taken up by the apoE or low density lipoprotein receptor (LDLR)) [13] on hepatocytes is an example of the latter strategy and was recently approved for clinical use (2018). More examples show the applicability of this approach [14].

Smaller drug targeting constructs, which in some definitions are also included among nanomedicines, instead make use of active targeting ligands to induce drug uptake in the target area or target cells. Such targeting ligands bind to a specific receptor on designated target cells. Obviously, nanomedicines using a combination of passive and active targeting are also highly investigated, although their success so far has been limited and has also been highly debated [15,16,17]. Active targeting approaches are mostly antibody-mediated targeting strategies but can also comprise the coupling of other targeting ligands such as lipoproteins, peptides, sugar moieties, aptamers and smaller epitopes.

The development of different nanoparticles as drug carriers has gained speed in recent years since Doxil approval, but the application of this concept in patients is still hampered by serious hurdles like for instance uptake by the reticulo-endothelial system [18] and corona formation that affect biodistribution and efficacy [19,20,21]. Moreover, the development of actively targeted drugs has seen tremendous growth in recent years, but most targets, that is, pathogenic cells that play a key role in disease progression, can still not be reached selectively by carriers. This review, outlining our own research activities of the past years and putting them in a broader perspective, will discuss our approaches to address both issues, while also presenting some of the current challenges in these applications.

Overall, both larger drug delivery systems and smaller targeted constructs constitute versatile platforms for multiple therapeutic entities. The requirements of such platforms may differ because both types of therapeutics are subjected to different clearance mechanisms and driving forces that affect their distribution in plasma, tissues and within cells. In all cases, the first step for the development of efficient drug delivery systems and targeted drugs is to obtain fundamental knowledge on these mechanisms and the existing driving forces.

## 2. Drug Targeting Constructs

**Figure 1:**
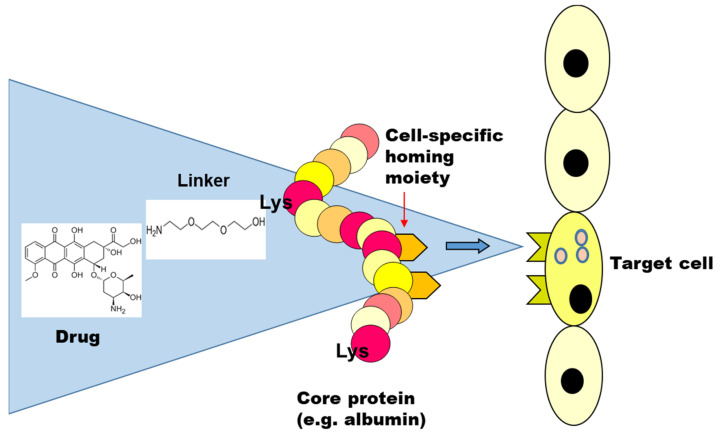
Attachment of homing devices and drugs to a monomeric core protein: general concept. The scheme illustrates the 4 elements constituting a drug targeting construct: the monomeric carrier, a homing receptor ligand (the targeting ligand), a therapeutic compound, and a linker between the drug and the carrier.

A simple drug targeting construct using a single, monomeric carrier represents, in most cases, the combination of four entities: the monomeric carrier, a homing receptor ligand, or targeting ligand, a therapeutic compound, and a linker between the drug and the carrier (Figure 1) [22]. These four elements represent four different challenges. The monomeric carrier, for instance, should be able to carry an effective payload [23], be non-immunogenic and exhibit the proper distribution profile, that is, preferably long circulating, yet with a good penetration into the target area. The linker should be stable enough to prevent release of drugs in plasma, yet biodegradable to ensure release of drugs at the target site [23,24]. The drug should be effective only after release from the carrier inside the cell and the homing device should be selective for the target receptor which in turn should preferably be unique for the designated target cell. In case of the use of large sized nanocarriers, therapeutic entities can be incorporated into the carrier without a linker, which is a huge benefit of such nanocarriers, but other challenges then emerge, which are reviewed below.

We illustrate challenges in the design of drug targeting constructs within the example of liver fibrosis, and present some of the solutions investigated in this area within our work. We focus on this example because in our view this represents a disease that cannot be cured without cell-selective drugs. Many experimental therapeutic compounds had beneficial effects in one hepatic cell-type yet displayed the opposite effects in the neighboring cell type (for review see [25]). For instance, use of apoptosis-inducing drugs to prevent proliferation of hepatic stellate cells has been proposed as anti-fibrotic strategy [26,27], yet survival and proliferation of hepatocytes is essential for liver regeneration [26,28]. Similarly, use of drugs to stimulate hepatocyte proliferation confers the risk of tumor formation while the use of anti-inflammatory drugs to prevent chronic inflammation that stimulates progression of fibrosis may inhibit the immunological response against hepatitis B and C viruses or gut-derived bacterial products [29,30] that stimulate progression of liver fibrosis [31,32,33,34]. Similar processes occur during fibrotic processes in other organs such as the heart, kidney, lung and intestines [35,36], and even during tumorigenesis [37,38], so our approach may also be useful to anticancer therapies in particular in types of cancers associated with significant stroma formation.

A key element in drug targeting ligands is the homing ligand. In our lab we tested sugars moieties (galactose, mannose and mannose-6-phosphate), charge modification (succinylation) and peptides (such as the PDGF receptor-recognizing peptide pPB (C*SRNLIDC*), or the tripeptide Arg-Gly-Asp, RGD) and we were able to selectively deliver drugs to all the different resident hepatic cells, i.e., hepatocytes, Kupffer cells, Hepatic stellate cells and endothelial cells [39]. An overview of this and the rationale behind different approaches is presented here.

## 3. Drug Targeting Approaches in Fibrotic Livers

Liver fibrosis is caused by chronic hepatocyte damage, generally followed by chronic inflammation, and tissue regeneration, characterized by excessive scar tissue formation [34,40,41,42]. In each phase of the disease, different cell-types play a key role and different types of drugs therefore need to be delivered to either hepatocytes, Kupffer cells or hepatic stellate cells and carriers to all of these cell types are available [22,39]

Galactose or the galactose-containing disaccharide lactose bind to the asialoglycoprotein receptor (ASGP-R) on hepatocytes [43] and coupling of these sugar moieties to several constructs led to selective uptake of these compounds in hepatocytes [44,45,46] or hepatocellular carcinoma [47]. In the past decades, this strategy has been applied for the delivery of many hepatoprotective drugs to these cells, and the benefits of selective delivery of anti-viral drugs using such carriers has been presented in recent publications. For an overview see Table 1, and for a specific review on Hepatitis B see [48].

Mannose moieties bind to the mannose-receptor Cluster of Differentiation 206, (CD206) on macrophages [49]. CD206 is a marker for M2 macrophages [50], which represents the pro-fibrotic phenotype of this cell type [50]. The polarization of macrophages into either a pro-inflammatory or a pro-fibrotic phenotype can greatly affect disease progression [34,36,51]. This receptor is therefore an appropriate target to deliver anti-fibrotic drugs to the liver (Table 1) and we therefore applied mannose-albumin constructs for the delivery of dexamethasone to Kupffer cells [52,53,54,55]. Kupffer cells stimulate fibrosis by the production of many pro-inflammatory mediators in response to damage or activation of other cells [56] and chronic activation of Kupffer cells is one of the main drivers of a perpetuating fibrogenic process [34,36,40,41]. A selective uptake of dexamethasone coupled to mannosylated albumin (dex-man-HSA) was demonstrated in Kupffer cells, with minor uptake in sinusoidal endothelial cells and no uptake in other cells [52]. However, when the effectivity was tested we found anti-inflammatory effects as well as pro-fibrotic effects of our constructs, yielding only a minor net anti-fibrogenic effect in animals with liver fibrosis [52]. We hypothesized that Kupffer cells play a dual role during fibrogenesis. This illustrates another benefit of drug targeting: selective delivery of drugs may yield more insight into the role of target cells in the pathogenesis of the disease. In our case, a shift from anti-inflammatory activities to pro-fibrotic activities of the target cell was noted [52], even before the discovery of M1 and M2 macrophages that have opposite activities during inflammation and fibrosis progression [34,36,51]. We also identified the key effector cell in Prostaglandin E2-induced activity of EPAC-1 (Exchange protein activated by cAMP 1) by targeting Prostaglandin E2 to different hepatic cell-types using different carriers and compare the effects [57]. In case of Kupffer cell targeting, it should be noted that delivery of large constructs to macrophages always confers the risk of direct activation of these cells by the carriers [52,58,59].

One of the other key players in liver fibrosis is the hepatic stellate cell (HSC) [40,41,60,61], and we were the first to show that Mannose-6-phosphate-(M6P)-albumin is taken up by the Insulin-like Growth Factor II/M6P receptor expressed on activated HSC [62]. Upon activation induced by liver damage or activation of other hepatic cells, resting HSC acquire capabilities to migrate to the site of damage and proliferate, amongst others, by expressing growth factor receptors [40,41,60,61]. Subsequently these cells produce extracellular matrix constituents such as collagen I and III. The IGFII/M6P (insulin-like growth factor type 2/mannose-6-phosphate) receptor on this activated cell type could be targeted by coupling at least 21 molecules mannose-6-phosphate to albumin [62]. We found rapid binding and uptake in vitro in cultures of activated fibroblasts and HSC-selective uptake in vivo in animals with liver fibrosis [62]. In addition, we developed a cyclic peptide that binds to the Platelet Derived Growth Factor β-receptor (PDGFβR), referred to as pPB [63], and a cyclic peptide against the collagen type VI receptor [64]. After coupling to a core molecule both cyclic peptides selectively accumulated in activated HSC. For pPB it was demonstrated that at least two peptides are required to achieve binding to the dimeric PDGF-βR [63]. In particular the pPB-based carrier and the M6P-based carriers have been extensively used for the delivery of many antifibrotic compounds to this cell-type including anti-proliferative drugs (doxorubicin [65], mycophenolic acid [66]), apoptosis-inducing drugs (gliotoxin [67], 15-d-Prostaglandin J2 [68]), anti-inflammatory drugs (pentoxifyline [69], Interleukin 10 [70], Prostaglandin E2 [57]), collagen synthesis inhibitors (an ALK5 inhibitor [71]) a Rho-kinase inhibitor (Y27632 [72,73,74,75]), a tyrosine kinase inhibitor (Imatinib [76]) and other inhibitors of HSC activation (the Angiotensin Receptor I antagonist Losartan [77]. These are listed in Table 1. Cell-selectivity and organ specific uptake of all constructs was demonstrated in vivo in animal models and all compounds displayed therapeutic effects. In most cases the targeted compound was significantly more effective than the untargeted native compound. For several compounds it was also shown that adverse effects of drugs were strongly reduced in animals with liver fibrosis compared to the untargeted compound [75,78,79].

Kinase inhibitors such as Imatinib and Y27632 are very relevant candidates for cell-specific delivery because they are effective in the treatment of liver fibrosis or portal hypertension associated with this disease [73,80,81], yet they have many adverse effects [82]. However, the general chemical structure of all kinase inhibitors [83] formed a challenge to couple it to proteins because no reactive groups are available. We therefore applied a platinum-based linker between –SH groups in the protein and nitrogen in the aromatic ring of kinase inhibitors [69]. This formed a coordinated bond that allowed coupling of kinase inhibitors. A slow release, up to 7 days, of the inhibitors from the carrier was found, induced by high intracellular glutathione (GSH) concentrations, creating the benefit of a slow-release depot within the target cell [69]. This is particularly relevant for the treatment of chronic diseases. These studies may therefore provide important information for the chronic use of kinase inhibitors which play a pivotal role in current therapies against cancers and chronic inflammatory diseases [84,85,86].

The constructs described above are generally rapidly taken up via receptor-mediated endocytosis which is the preferential route for therapeutic entities that can escape from endosomes (lipids) or are non-degradable, like most small chemical entities. However, biologicals such as cytokines and larger proteins may lose their therapeutic effectivity via this route. Cytokines are very potent entities that are active in the pico-molar range and affect disease activity at many different levels, so such compounds are very promising, yet they are also associated by multiple adverse effects. Their therapeutic use may greatly benefit from cell-specific delivery. For this reason we particularly focused on the cell-specific delivery of cytokines and examined strategies to deliver these potent biologics to target receptors without losing effectivity.

To that end, our peptide that binds to the PDGF-β-receptor (pPB) [63] was used to deliver Interferon γ to HSC. Increased PDGF-β-receptor expression is a hallmark of HSC activation and fibrogenesis and Interferon γ (IFNγ) has antifibrotic activities in HSC [87,88,89], yet its potent pro-inflammatory activities prevent its clinical use [90]. Indeed, we found selective binding and uptake of pPB coupled to IFNγ in activated fibroblasts in fibrotic livers [91,92], but also in fibrotic kidneys [93] and in stromal cells of tumors [94]. In these studies antifibrotic effects of IFNγ were potentiated and adverse effects were reduced or even absent. In subsequent studies we removed the IFNγ-receptor binding site of IFNγ to prevent uptake via this receptor and coupled pPB to the truncated cytokine to induce binding to the PDGFβR [95,96]. This minimized construct was also very effective. Furthermore, the delivery of Interleukin 10 to the M6P/IGFII receptor on HSC may be an effective strategy to re-direct this cytokine [70]. These compounds represent therefore an interesting therapeutic entity to treat fibrotic diseases, yet the payload should be increased to achieve optimal effects.

An increased payload may be achieved by applying nanocarriers with larger size that can accommodate larger concentrations of compounds or—on the opposite side—by a significant reduction of the molecular weight of the constructs. As outlined above, we tested both options by creating a peptidomimetic IFNγ molecule [95,96], combined with just one bicyclic-pPB molecule, and a small linker (polyethylenglycol, PEG, of 2kd) to create stability. The molecular size of this construct is only 9 kD, similar to insulin, and significantly lower that our earlier constructs with albumin and native IFNγ (>150 kD). This small compound is now tested for diagnostic purposes in patients with liver fibrosis (see www.biorion.com, accessed on 23 December 2021).

## 4. Other Monomeric Carriers

Albumin represents a very suitable drug carrier because it is very versatile, is able to penetrate in fibrotic tissue even in end-stage human cirrhotic livers [53,122], and is non-immunogenic, although chronic treatment requires the use of mouse albumin instead of human albumin [123]. It is biodegradable and long circulating due to its size of 67 kD, which is just above the cut-off for glomerular filtration (approximately 65 kD). Yet, it requires chemical coupling of drugs, which is not always possible, and the payload may be too low for some drugs, for review see [22,25]. However, all homing devices described here can also be incorporated in larger nanoparticles. We have demonstrated this by using liposomes [124,125,126,127], and viral vectors [128], but many other options are available [129,130], although tissue penetration and exploiting the EPR-effect in fibrotic tissue is difficult for larger nanomedicines [131].

Overall, the examples discussed illustrate the importance of understanding the details of the mechanism by which targeted drugs are processed in vivo as well as at cellular level and intracellularly. With such knowledge, the design of small targeted constructs capable to deliver their drug load to the target cell can be optimized as in the examples discussed.

## 5. Nanomedicines to Delivery Drugs into Target Cells

**Figure 2:**
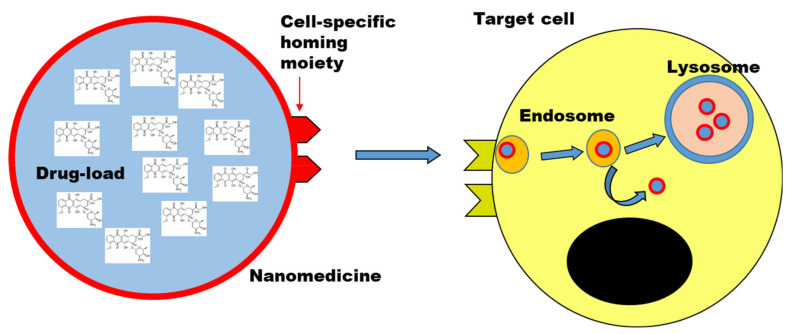
Nanomedicine to deliver drugs to target cells. Scheme illustrating a nanomedicine carrying a high-load of drug molecules to a target cell. Drug targeting strategies can be combined by decorating the nanomedicine with targeting ligands (cell-specific homing moiety). Upon uptake by cells, typically by endocytosis, nanomedicines are trafficked along the endo-lysosomal pathway, unless designed to avoid it (i.e., endosomal escape strategies). Objects not to scale.

In comparison to the simpler targeted drug constructs, larger nano-sized materials (typically in the range of tens to few hundreds nanometer) can be loaded with higher amounts of drugs and be used as a drug carrier to improve the delivery to the target cells. Nanomedicines can be made from multiple types of materials, including lipid-based and polymer-based nanomedicines, bio-inspired materials (including for instance viral vectors and natural nanoparticles such as exosomes and lipid nanoparticles), and inorganic nanoparticles [132]. Figure 2 shows a simplified scheme of a nanomedicine. Active targeting can be coupled to the drug carrier by decorating the nanomedicine with targeting ligands.

Although quite different in their structure, several important features are common between smaller targeted constructs and these larger drug carriers, and the way they are processed at tissue and cell level. A key aspect in comparison to small molecular drug compounds is that these advanced drug delivery systems are too large to simply diffuse into cells, rather they are internalized by cells via active mechanisms of endocytosis which require cell energy, and are then processed by cells into specific locations (usually the lysosomes, see later for further details), instead of partitioning based on their solubility [133,134]. This is also one of the reason why sometimes they are both classified as nanomedicines, in order to stress the common aspects in the way they are processed because of their (larger) nano-scale size.

In order to achieve efficient targeting and drug delivery, a better understanding of how these objects are processed at organism and at the cell level is required, so that their design can be changed in order to control their behavior and efficacy. We discuss in the next sections some key aspects that need to be understood and controlled towards this aim.

## 6. Controlling the Interactions of Targeted Drugs and Nanomedicines with Cells

### 6.1. Interactions with Biological Fluids

Another key aspect which is common for targeted drugs and drug carriers is that once they are applied in biological environments, they can be modified by adsorption of biomolecules on their surface. A classic example is the binding of opsonin proteins in blood, which induces recognition by the cells of the immune system and removal from circulation [135,136,137]. More recently, the layer forming on drug carriers and targeted drugs upon interactions with biological fluids has also been referred to as “the biomolecule corona” [20,138]. Protein binding and corona formation are a common barrier to drug delivery which affects both targeted constructs and larger nanomedicines. Not only opsonization can activate clearance by immune cells, but protein binding can also mask targeting ligands thus impairing their interactions with the targeted receptors. For instance, we found that in vitro transferrin-targeted nanoparticles lost their capacity to interact with the transferrin receptor upon addition of serum in the medium and corona formation [139].

Several strategies have been developed to reduce protein binding in biologic fluids, including the use of antifouling polymers and surfaces, and the introduction of spacers between the therapeutic compound or drug carrier and the targeting ligand in order to maintain its capacity to interact with the targeted receptor, even after corona formation [135,140]. Polymers such as PEG (polyethylene glycol) are often used for both applications [141]. Other bio-inspired strategies to reduce clearance are also emerging, such as the use of marker-of-self and don’t-eat-me signals to decorate nanomedicines and camouflaging them from the immune system. Examples include modification with CD47 or coating of drug carriers with cell membranes from leukocytes or erythrocytes [135,142,143,144].

At the same time, it has also emerged that the adsorbed biomolecules may mediate interactions with specific cell receptors and in this way, they can also drive nanomedicine distribution in vivo. For instance, vitronectin in the corona was found to increase uptake via ανβ3 integrin receptor in vitro and in vivo [145,146]. Similarly, it was suggested that apolipoprotein E in the corona facilitates nanoparticle transcytosis across the blood brain barrier [147], while we and others found that silica nanoparticles are internalized by cells upon interaction of apolipoproteins in their corona with the low density lipoprotein receptor, LDLR [148,149]. Interestingly, this specific interaction was observed only when forming a corona using high serum concentrations, mimicking in vivo blood plasma concentration. This kind of observations challenges the use of standard low concentration of serum or also artificial serum-free conditions when testing nanomedicines and targeted drugs in vitro. Thus the proteins adsorbed on the nanomedicine can enable new ways to achieve targeted uptake into specific cells, and similar effects have been observed also in vivo. An example of this is that of Onpattro, a lipid nanoparticle carrying small interfering RNA (siRNA) and which – as mentioned previously- adsorbs apoE from plasma, thus promoting uptake into the liver hepatocytes via the LDLR [11].

On the opposite side, the binding of other proteins known as “dysopsonin” proteins, such as for instance albumin, can prolong plasma residence time [150,151]. Similarly, it has been reported that certain corona proteins adsorbed on nanomedicines in blood can also be read by immune cells as a don’t-eat-me signal. An example of a corona protein associated with reduced uptake by cells via this kind of mechanisms is clusterin [152]. Interestingly, the authors identified this protein as a major component in the corona forming on PEGylated nanoparticles and suggested that it is this specific protein in the corona to confer the so-called PEG “stealth-effect”, as opposed to the overall reduced protein binding. Recently reduced uptake has been observed also for nanoparticles with a corona rich in histidine rich glycoprotein [153,154]. Interestingly, reduced uptake could be achieved also when pre-coating nanoparticles with an artificial corona of histidine rich glycoprotein [153]. Thus, the corona may be engineered prior to administration to promote the desired interactions. Based on similar observations, more studies are focusing on the screening of the corona forming on panel of nanoparticles as a novel tool to identify molecules that can promote or reduce uptake by cells [155,156,157,158]. We performed similar studies using silica nanoparticles of different size and surface functionalization, as well as liposomes of different charge which showed very different uptake levels by cells [153,159]. Similarly, we have used a panel of different nanoparticles thus different corona to identify receptors involved in nanoparticle uptake into endothelial cells from different organs (Aliyandi et al. unpublished) [160].

Overall, these examples (which are summarized in Table 2) highlight that a better understanding of the complex interactions of nanomedicine in biological fluids and the effects of corona formation on distribution and uptake by cells is necessary for the design of successful targeted drugs and nanomedicines. At the same time such knowledge can help to discover novel strategies for targeting and to prolong nanomedicine plasma residence time.

Ultimately, distribution, uptake in the targeted cells and clearance, all affect the therapeutic efficacy, thus the effects of corona formation on these aspects need to be carefully considered.

### 6.2. Interactions with Cells and Intracellular Fate

After administration and modifications in biological fluids, the next steps into the journey of nanomedicines and targeted drugs are the interactions at the cell membrane both of the targeted cells and all other cells, and the following uptake mechanism and intracellular trafficking.

While we discussed above examples of drug targeting strategies and effects of corona formation on targeting and interactions with cell receptors, there are other key aspects in the interactions at the cell membrane of nanomedicines and targeted drugs which need to be better understood and controlled in order to improve their efficacy.

One important aspect that has emerged is that even when specific receptors are targeted (via active targeting ligands or via the corona adsorbing on nanomedicines), the targeted drug or nanomedicine may be internalized and processed by cells in different ways comparing to what usually observed for the same receptors and their natural ligands [161]. For instance, we found that even when the LDLR mediates nanoparticle uptake, the mechanisms of internalization is not clathrin mediated, as usually observed for this receptor [148]. Similarly, nanoparticles targeting the transferrin receptor were not recycled and exported by cells as transferrin, but were trafficked by cells towards the lysosomes [139]. More efforts are needed to understand how after interactions with certain receptors, cells internalize and process these materials. Without this knowledge, the development of nanomedicines and targeted drugs risks to remain mainly limited to screening of multiple materials, as opposed to the tailored design of materials with the properties required to achieve the desired outcomes at cell level (as well as in vivo).

The mechanisms cells use for the internalization of nanomedicine and targeted constructs also affect uptake efficiency and intracellular trafficking kinetics [134,161]. These factors affect the time required to reach the therapeutic dose intracellularly, and the time required to reach specific locations inside cells, which all together contribute to the final therapeutic efficacy.

Characterizing the mechanisms by which nanomedicines and targeted drugs are internalized by cells remains highly challenging [134,161,162]. Many contrasting results are often reported and it is hard to draw conclusions on how nanoparticle properties affect the mechanism of uptake or how they vary among different cell types. This is also due to complicating factors such as corona formation, which are not always controlled and reproduced among different studies. Additionally, the methods usually applied to try to characterize these mechanisms are often limited [134,162]. We have shown examples of this for classic transport inhibitors commonly used for this purpose [163]. For instance we found that when using high concentration of inhibitors such as chlorpromazine, a common drug to block clathrin-mediated endocytosis, reduced nanoparticle uptake was observed but this was due to strong toxicity on cells, as opposed to a specific inhibition of the pathway. Additionally, many of these compounds lost their efficacy when applied to cells in a medium with serum, probably due to protein binding [163]. Given the effect of corona formation on targeting and nanomedicine interactions with cells, thus the need to include serum when testing nanomedicine and targeted drugs in vitro, other compounds whose efficacy is not affected by the presence of serum need to be used [161,163]. Thus, without controls for similar effects and to exclude toxicity, results obtained with these common transport inhibitors may lead to wrong conclusions. Given these limits, multiple methods need to be combined. Furthermore, we cannot exclude that nano-sized materials may be processed in different ways than natural ligands and alternative endocytic mechanisms not yet characterized may be triggered by these special cargoes. Thus, new methods need to be developed to identify potential novel targets not yet associated to cell uptake mechanisms. To this aim, we have applied genome-wide screening and developed proteomic based approaches to identify all genes and proteins involved in nanoparticle uptake (Montizaan et al., unpublished; Garcia-Romeu et al., unpublished). Indeed, many novel targets have been identified thanks to this kind of approaches and current efforts are focused on understanding their role.

Finally, it is commonly observed that after uptake most nanomedicines are trafficked by cells along the endo-lysosomal pathway towards the lysosomes [133,164]. While this can be exploited when these are the targeted intracellular compartments, all drugs which require accumulation into other locations inside cells require to escape the endo-lysosomal compartments or are otherwise degraded once they are transported in the lysosomes. Several strategies for endosomal escape have been investigated, and often are limited by toxicity and poor efficacy [165,166]. In the case of RNA delivery and recently approved lipid nanoparticles such as Onpattro or the RNA vaccines against COVID-19, this has been achieved utilizing ionizable lipids which become positively charged only once inside the slightly acidic environment of the endosome [11,12]. The acquired cationic charge can destabilize the endosomal membrane and promote escape of the nanomedicine into the cytosol. This could be developed by carefully tuning lipid design to change their pKa in order to promote this effect.

This is another example which beautifully illustrates the importance of understanding how nanomedicines and targeted drugs are processed by cells so that their design can be carefully tailored in order to achieve the desired effects.

In order to characterize the intracellular trafficking of nanomedicines and targeted drugs, methods to be able to follow them inside cells over time and determine their intracellular location as well as the time needed to reach certain location are needed. For instance, we have used live cell fluorescence microscopy imaging to measure nanoparticle location inside cells over time and in particular their colocalization with the lysosomes [167]. Using nanoparticles of different sizes and theoretical modeling, the intracellular trafficking kinetics could be determined, thus how nanoparticle size affect the departure time from the cell membrane and arrival time to the lysosomes. With similar methods, we can then determine how nanoparticle properties affect these details of intracellular trafficking, which also affect nanomedicine efficacy. Our results also showed that within the same cells some nanoparticles arrive to lysosomes within short times, while others take much longer or get stuck somewhere else and never seem to arrive there. Additionally, we also found that not only uptake efficiency, but also intracellular trafficking kinetics strongly vary within individual cells in a cell population [167]. Clearly, further studies are needed to understand similar observations and learn how to design nanomedicines and targeted drugs with controlled and uniform behavior at cell level. Within this context, in order to try to understand the sources of such heterogeneity in nanoparticle uptake by cells, we recently used repeated cell sorting to isolate cells with low and high nanoparticle uptake within a cell population and used transcriptomics to identify differences in gene expression in such sub-populations [168].

Next to such imaging-based approaches, taking advantage of latest developments in high sensitivity flow cytometry, we have also developed a method based on organelle flow cytometry to gain temporal as well as spatial information on nanoparticle intracellular distribution and trafficking [169]. The method allows to gain information on nanoparticle intracellular location as usually obtaining by imaging-based methods but with the high throughput and robust quantification enabled by flow cytometry. Thus, cells are exposed to nanoparticles, then all organelles are extracted and characterized by flow cytometry. In this way, intracellular trafficking kinetics can be easily obtained from thousands of organelles extracted from thousands of cells, without the complex analysis required to extract similar results from imaging-based methods. The two different methods present different advantages and limits, thus they can be used to complement each other in order to gain a better understanding on the details of intracellular trafficking of these object by cells. A better understanding of how cells process nanomedicines and targeted drugs is needed in order to optimize their design and efficacy.

## 7. Concluding Remarks

Many new therapeutic entities with a beneficial pharmacological activity profile have an unfavorable distribution in the body, leading to toxicity or poor therapeutic effects. In fact, more than 90% of all new active substances that enter clinical phase I fail in subsequent clinical trials [170] and 79% of these failures are due to safety or efficacy issues [170]. The development of generic versatile drug delivery systems may therefore have profound effects in the pharmaceutical world and medicine. It may prevent the rejection of many potent drugs, and thus prevent loss of time and money. In parallel, it may open new opportunities for the treatment of cancer, chronic regenerative diseases, infections and other serious diseases with an unmet medical need.

An important group of drugs that may benefit from advanced delivery systems are biologics. Approximately 80% of all new biologics approved by the FDA (Food and Drug Administration) in 2020 are monoclonal antibodies [171], and they represent 20% of all new FDA approvals. These can be considered an advanced drug delivery system in itself. However, many other biological compounds such as antisense oligonucleotides, RNA-and DNA-based therapeutics, peptides and proteins, will greatly benefit from a delivery system, not because of toxicity but in order to have a beneficial effect at all. Most of these more complex drugs have poor bioavailability and can easily be degraded. Additionally, they are too large to simply diffuse into cells, and are often poorly internalized, thus require a drug carrier for efficient internalization. More and more of these types of compounds, including peptides and oligonucleotides referred to as TIDES [172], emerge in the list of FDA-approved molecules. They already represented 10% of all FDA approved drugs between 2016 and 2020 [172], underlining the need for further development of nanomedicines and drug targeting strategies. The recently developed RNA and DNA vaccines against COVID-19 infection have illustrated the potential of such novel types of drugs enabled by nanomedicine [173,174].

Finally, nanomedicines as well as a drug targeting open many possibilities for a theranostic approach by delivering a diagnostic ligand and a therapeutic compound to the same cell or target site [175]. Theranostics allow stratifications of patient groups before treatment and make early detection of disease activity and assessment of therapeutic efficacy of experimental drugs possible, which are all key hurdles in clinical trials, in particular for chronic fibrotic diseases [176]. This type of personalized medicine may therefore represent an important innovation in medicine in the future.

## Figures and Tables

**Table 1:** Overview of the different target cells and target receptors in the liver that have been applied for the delivery of drugs in animals models of disease.

Target Cell	Target Receptor	Homing Ligand	Delivered Drug	Reference
Hepatocytes	Asialoglycoprotein-receptor (ASGP-R)	Galactose or Lactose	Antiviral Antitumor Hepatoprotective	[44,45,46]
	Low-density lipoprotein Receptor (LDL-R)	APO-E	RNA-based drugs	[97,98,99]
	Coxsackie and adenovirus cell adhesion receptor (CAR)	Adenoviruses Adenoviral-derived ligands	Genes	[100]
Macrophages	CD206	Mannose	Anti-inflammatory	[52,53,57,101]
Hepatic Stellate Cells and Myofibroblasts	Platelet Derived Growth Factor β-receptor (PDGF-β-R)	pPB peptide	Antifibrotic Anti-proliferative Anti-inflammatory Pro-apototic Rho-Kinase inhibitors Collagen synthesis inhibitors Tyrosin-kinase inhibitor Angiotensin inhibitor	[57,63,68,72,102]
	Insulin-like-Growth Factor II receptor (IGFII-R)	Mannose-6-phosphate	Anti-fibrotic	[65,68,71,73,103,104,105,106,107,108]
	Vitamin A-receptor	Retinoic acid	Anti- collagen chaparone glycoprotein (gp46)- siRNA	[99,109,110,111]
Progenitor cells/Cholangiocytes	Integrin Avβ6-receptor	αvβ6 ligand/antibody		[112,113,114]
Endothelial Cells	Scavenger receptor	Succinylated molecules	Anti-inflammatory	[54]
	Integrin receptor	RGD-peptides	Antiangiogenic Anti-inflammator Kinase inhibitors	[115,116,117,118]
	Hyaluronic Acid-recptor	Hyaluronic acid		[119,120,121]

**Table 2:** Examples of effects mediated by corona biomolecules adsorbed on nanoparticles and other nanoparticle modifications affecting corona formation. The Table summarizes some examples of the effects mediated by the corona forming on nanoparticles which have been described in literature and which are discussed in Section 6.1. We stress that the Table is not complete and just includes some selected examples as a reference.

Nanoparticle Modification or Corona Component	Effect Reported	Selected Examples
Corona formation	Can mask targeting ligands in vitro	[139]
Opsonin proteins in the corona	Activation of immune cells, nanoparticle removal from circulation	[135,136,137]
PEGylation	Reduced protein adsorption and/or binding of dysopsonin proteins in the corona such as clusterin	[135,141,152]
Dysopsonin proteins in the corona	Prolonged circulation time	[150]
Albumin in the corona	Prolonged circulation time	[151]
Clusterin (apolipoprotein J) in the corona	Prolonged circulation time	[152]
Histidine rich glycoprotein in the corona	Prolonged circulation time	[153,154]
CD47 functionalization	Marker of self, “don’t eat me” signal for immune cells, prolonged circulation	[143]
Leukocytes cell membrane coating	Nanoparticle camouflage, prolonged circulation and increased accumulation in inflamed areas	[142]
Red-cell membrane coating	Nanoparticle camouflage, prolonged circulation	[144]
Apolipoprotein B in the corona	Uptake mediated by LDLR	[148,149]
Apolipoprotein E in the corona	In vivo targeting of liver hepatocytes via LDLR	[11]
Apolipoprotein E in the corona	Promotes nanoparticle transcytosis across the blood brain barrier	[147]
Vitronectin in the corona	Increased uptake via ανβ3 integrin receptor in vitro and in vivo	[145,146]
